# On the total albumin losses during haemocatharsis

**DOI:** 10.1007/s10047-023-01430-y

**Published:** 2024-01-18

**Authors:** Anastasios J. Karabelas

**Affiliations:** Chemical Process and Energy Resources Institute, Centre for Research and Technology-Hellas, 6th Km Charilaou - Thermi Road, Thermi - Thessaloniki, GR 57001 Greece

**Keywords:** Haemocatharsis, Albumin loss, Permeation to dialysate, Secondary membrane formation, Protein conformational changes

## Abstract

Excessive albumin losses during HC (haemocatharsis) are considered a potential cause of hypoalbuminemia—a key risk factor for mortality. This review on total albumin losses considers albumin “leaking” into the dialysate and losses due to protein/membrane interactions (i.e. adsorption, “secondary membrane formation” and denaturation). The former are fairly easy to determine, usually varying at the level of ~ 2 g to ~ 7 g albumin loss per session. Such values, commonly accepted as representative of the total albumin losses, are often quoted as limits/standards of permissible albumin loss per session. On albumin mass lost due to adsorption/deposition, which is the result of complicated interactions and rather difficult to determine, scant in vivo data exist and there is great uncertainty and confusion regarding their magnitude; this is possibly responsible for neglecting their contribution to the total losses at present. Yet, many relevant in vitro studies suggest that losses of albumin due to protein/membrane interactions are likely comparable to (or even greater than) those due to leaking, particularly in the currently favoured high-convection HDF (haemodiafiltration) treatment. Therefore, it is emphasised that top research priority should be given to resolve these issues, primarily by developing appropriate/facile in vivo test-methods and related analytical techniques.

## Introduction—scope

In this review, the generic term HC (haemocatharsis) is used to designate all currently employed modes (i.e. haemodialysis, haemodiafiltration, haemofiltration). In recent years, HC modes relying more on convection than on diffusion, are preferred for treatment of ESRD (end-stage renal disease) patients. Many studies (e.g. [1–3]), show that, in particular OL-post-HDF (online, post-dilution haemodiafiltration), involving large transmembrane/ultrafiltration rate (and the concomitant large substitution volume), is quite beneficial leading to reduced mortality [1, 3]. However, there is concern that, under such conditions (involving relatively high-permeability membranes), excessive albumin losses may occur, which are possibly associated with hypoalbuminemia, at least for some ESRD-patient categories [4]. In view of these concerns, the total albumin loss, in grams per HC session, is commonly a criterion (i.e. a limit not to exceed) to take into account in assessing the overall performance of a particular HC protocol (including membrane type) in clinical practice. For instance, the Japanese Society for Dialysis Therapy (JSDT) has recommended such a standard/limit on permissible total albumin loss [5].

The literature at present is confusing, regarding the safe limit of albumin losses per session and how to determine it. There are recent papers recommending such a limit, using results of clinical studies, where only albumin losses to dialysate are considered (e.g. [4, 6, 7]). However, several other studies conclude that it is impossible at present to recommend such limits/standards for various reasons (e.g. [8, 9]). In parallel, extensive relevant work is performed on the undesirable effects due to interaction of plasma proteins with the HC membranes (i.e. their haemocompatibility) [10–12]. However, data obtained from such studies have been inadequately utilised to address the issues related to albumin losses. Therefore, in this paper, reviewing mostly recent relevant literature, an effort is made to shed light on key issues related to total albumin losses during HC, thus enabling prioritisation of research to improve the situation. In the following, factors and basic conditions favouring albumin losses will be outlined first; next, the status of literature on the types of potential losses will be separately dealt with.

## Albumin losses during haemocatharsis

### Types of losses: driving forces, conditions

There are two main types of albumin losses during HC, i.e*.* (i) albumin “leaking”/permeating into the dialysate and (ii) losses through albumin interaction with, and adsorption/deposition into/on, the HC membranes [13, 14]. Two basic driving forces and their rather complicated interaction are responsible for such losses. The main driving force for albumin permeation through the membranes is the effective (local) transmembrane pressure difference (leading to ‘ultrafiltration’ flow), along the hollow-fibre membrane filter, and to a much lesser extent (considered relatively insignificant) the protein concentration difference between plasma and dialysate, i.e. the mechanism of convection is dominant/controlling (over diffusion) in this case [2, 15]. In parallel, the physico-chemical interaction of plasma proteins (including albumin) with the polymeric (porous) material of hollow-fibre membranes determines the albumin adhesion into the pores and on the inner surface of HC hollow-fibre membranes as well as the possible formation of a protein layer on the membrane surface, through further deposition of albumin and other proteins (e.g. [14, 16]). This deposit layer, whose significance has been recognised long ago [13, 17], is often referred to as “secondary membrane” or fouling/’gel’ layer [17]. As noted below, the physico-chemical protein/polymeric-material interactions, also significantly affect albumin leaking to dialysate, due to the reduced membrane porosity and permeability caused by the adsorbed/deposited proteins [3, 15, 18]. In addition, it should be stressed that protein adsorption and fouling-layer/gel formation, is related to other complicated phenomena, including albumin unfolding/denaturation and ‘competitive adsorption’ among the most abundant proteins (i.e. albumin, fibrinogen, transferrin) onto the membrane, which have been studied mostly in vitro (e.g. [19]).

**Fig. 1:**
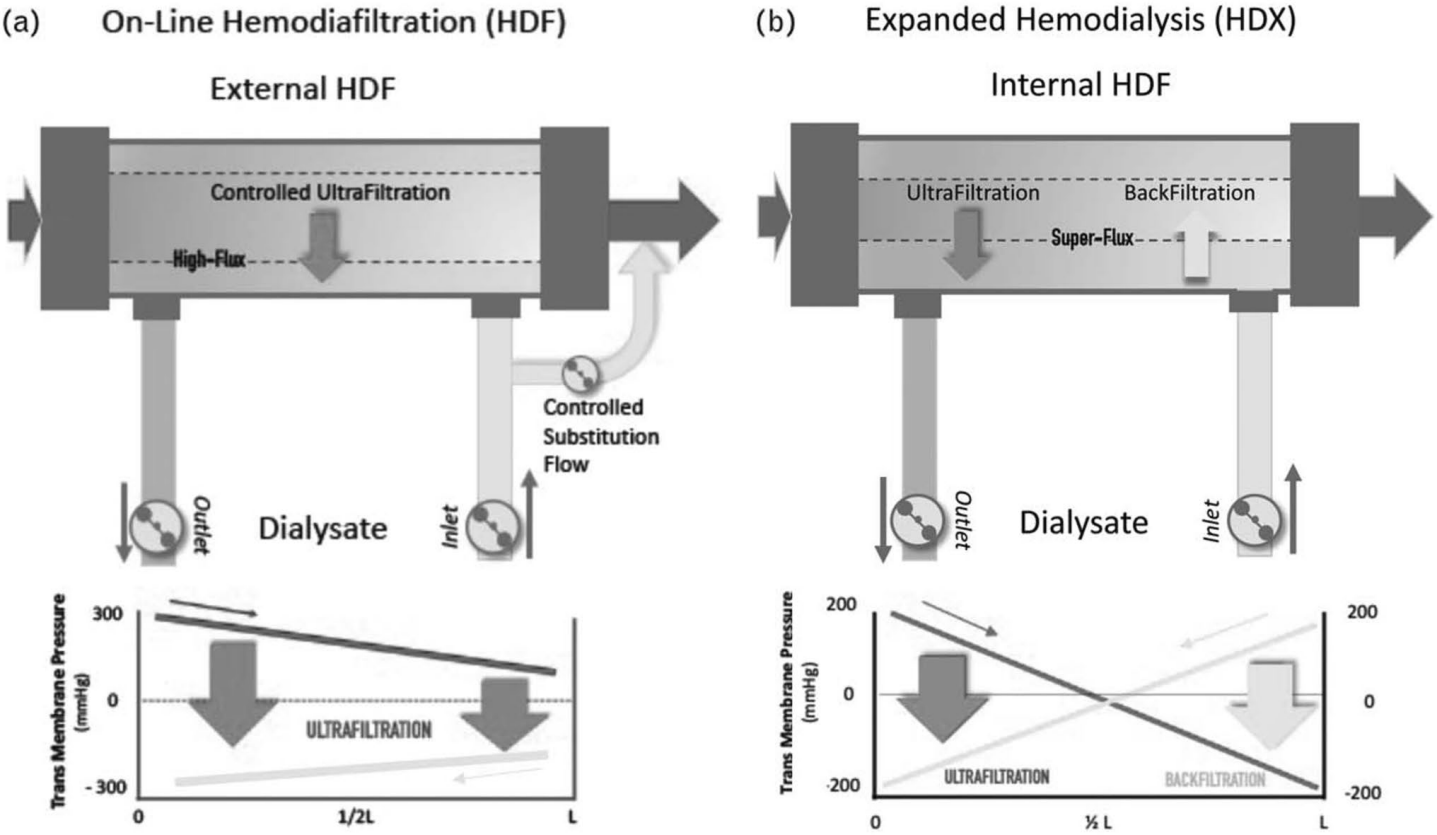
Principle and pressure profiles of convection-based therapies employing high-flux capillary membrane HC filters and ultrapure dialysis fluid. **a** *On-line haemodiafiltration* (post-dilution) with external fluid substitution. **b** *Expanded haemodialysis*, involving forced internal filtration, i.e. ultrafiltration and back-filtration. From Ref. [2] (with author’s permission)

In respect of blood fluid-dynamics, the most extreme conditions (favouring losses) are those prevailing during OL-post HDF (Fig. 1, [2]) for the following reasons. Due to the required large substitution volume, the total ultrafiltration rate (*Q*_UF_) in the HC filter is typically at high level, of order ~ 100 mL/min. Importantly, in OL-post HDF, there is only forward ultrafiltration along the entire HC filter (Fig. 1a) and no “back-filtration” as in other modes (Fig. 1b) [2]. Therefore, the local rate of permeating ultrafiltrate is unidirectional (Fig. 1a) and relatively high, favouring albumin leaking to dialysate. In addition, the inlet blood flow rate (*Q*_bin_), usually 300 mL/min, and the shear stresses at the membrane surface, are substantially reduced, leading to significant increase of albumin concentration and blood/plasma viscosity [15]. As is well known, these conditions favour protein/albumin deposition and “gel”/fouling-layer formation [14, 15].

To understand adhesion affected by membrane physico-chemical properties, very extensive work has been done on protein/membrane–material interaction, while pursuing haemocompatibility of materials for medical applications [10, 12]. In addition to issues related to HC hollow-fibre/membrane geometric features (fibre thickness, diameter), porous structure, surface porosity and roughness, emphasis is currently placed on specific physico-chemical properties, including hydrophilicity, electric charge and surface active units/species [20, 21].

### Issues regarding determination of albumin losses in vivo

The state of our knowledge on total albumin losses during HC is greatly affected (in fact shaped) by the relative ease, or (conversely) by the inherent/practical difficulties, of accurately determining (particularly in vivo) either type of albumin losses. Indeed, albumin permeating into the dialysate is fairly easy to directly and accurately determine (during, and at the end of, a HC session), through dialysate sampling/analysis and measurement of the exiting/disposable total dialysate volume. On the contrary, in vivo determination of albumin losses due to adsorption/deposition (based on periodic inlet and outlet blood samples) necessitates several steps, including accurate inlet and outlet blood flow-rate (*Q*_bin_, *Q*_bout_) measurements, serum/plasma separation and then analysis/characterisation of plasma samples for determination of albumin concentration. Such steps, necessary to determine small differences in albumin concentration between inlet and outlet streams, are marred by inherent uncertainties and experimental errors. It is noted that the plasma volume is changing along the HC filter due to ultrafiltration [2, 15] and likely during the 4-h HC session. In addition, the usual analytical errors and uncertainties of determining albumin in the plasma (at rather high concentrations) may be of the same order of magnitude as the changes/differences of interest, i.e. due to losses in the HC filter. Finally, it should be stressed that (in a series of ‘instantaneous’ periodic samples, taken throughout a session), such relatively small changes in albumin concentration are important as they contribute additively to the total albumin losses in 4-h sessions.

Another type of ex situ indirect determination of deposited/adsorbed albumin mass in used HC filters (right after a session or test) is employed, aiming to determine the deposits on/in the hollow-fibre membranes. Results of such experimental studies have been reported using modules after in vitro tests (e.g. [22]); however, very few test results are available with “fouled” HC filters after a session (e.g. [23]), as outlined below.

## Studies on quantification of albumin losses

### Albumin permeating into dialysate

Many clinical studies have been reported, involving ESRD patients, on albumin permeating into the dialysate. Only recent relevant publications are reviewed chronologically here.

Tsuchida and Minakuchi [24] in a clinical study involving 118 patients, (treated with a highly permeable HC filter), found that albumin leakage was on average 7.7 ± 1.0 g/session, whereas 314 patients using conventional high-flux HC filters exhibited lower albumin loss. They also reported that in the recommended Japanese standard for classification of HC membranes, “the desirable albumin leakage per treatment is less than 4 g”. In a clinical study by Fournier et al. [25], 8 patients underwent OL-post HDF and only albumin losses in dialysate were determined (i.e. 3134 ± 2450 mg/session); it was also reported that such losses did not lead to hypoalbuminemia. Although mass of albumin adsorption/deposition on the membrane was not determined, it was considered substantial depending on HC filter type. Vega et al. [26] in a cross-sectional study, involving 20 patients receiving OL-post HDF, analysed albumin leakage during the first hour of HC session. Moreover, ‘protein cake’ formation was considered responsible for the gradual reduction of permeability and albumin losses during this period. Potier et al. [7] collected data on albumin loss (to dialysate only) from sessions involving 37 patients and 19 different dialyzers; among other results obtained, they concluded that 4/19 dialyzers lose more than 5 g/session albumin and should not be used in OL-post HDF. Gayrard et al. [23], in a study involving 12 ESRD patients, determined total protein removal to dialysate ~ 2.3 g per session, in maximum convection OL-post HDF. They also employed a protein elution protocol ex situ to identify (and determine the mass of) particular plasma proteins (including albumin) adsorbed on the used HC filters. They reported that the total amount of adsorbed proteins in the membranes was only 6.1% of the respective amount of proteins removed through the dialysate*.* However, the accuracy/reliability of determining the total mass of the deposited proteins with their elution protocol is unclear, as discussed in “Discussion”.

Cuvelier et al. [27] reported on the case of a woman treated with high convective volume OL-post HDF, who developed severe hypoalbuminemia, attributed to massive albumin loss into dialysate, i.e. 23.6 g albumin loss in one session, whereas she only lost 4.6 g in a regular HD (haemodialysis) treatment. Such loss per session (23.6 g) is by far the greatest ever reported “leakage”, raising questions regarding its reliability/representativeness. Finally, in a recent study [28] involving 52 patients undergoing high-volume OL-post HDF, the temporal variation of albumin removal only through leaking was determined for three types of HC filters. Modest cumulative removal (~ 1.0 g to ~ 1.5 g per 4 h session) was reported. Moreover, “secondary membrane” formation was considered to interpret these data, although no attempt was made to quantify it. It was also concluded (with insufficient justification) that the albumin sieving-kinetics data point to reduced formation of ‘secondary membrane’.

Several recent review papers (presented chronologically) also deal with these issues. Boschetti-de-Fierro et al. [6] assessed clinical studies on the performance of HC filters. Although the significance of ‘secondary membrane’ was discussed, no such albumin loss data were provided. Furthermore, they suggested, based only on albumin leakage data [24], that “7.7 ± 1.0 g/session is an estimate for a threshold of albumin removal (that could impact serum albumin levels), which should not be exceeded…”. Similarly, Van Gelder et al. [8] concluded that with convective therapies (OL-post HDF), albumin loss (through leakage only) is significant (range: 0.08–7 g per 4 h treatment); however, they also noted that the acceptable upper limit of dialysis-related albumin loss remains unknown. Ward et al. [4] reviewed studies regarding the possible effect of albumin losses (only due to leakage) on hypoalbuminemia and the importance of concomitant inflammation on outcomes in ESKD patients. They cautioned on use of membranes causing albumin loss of 20 g/week, whereas the use of HC filters resulting in weekly loss of 12 g (i.e. ~ 4 g per session?) appeared to pose little risk to patients. Kalantar**-**Zadeh et al. [9] considered that albumin loss into the dialysate was (a potentially modifiable) cause of hypoalbuminemia; however, they also remarked that protein adsorption to the membrane and tubing can occur and that patients tend to lose approx. 6–8 g of total amino acids per session. In addition, it was noted that no definition of “excessive” albumin loss during dialysis has been proposed or accepted.

### Albumin–membrane interaction and adsorption/deposition

#### Competitive protein adsorption and albumin structural changes

The interaction of human plasma proteins with membrane materials has been extensively studied (in vitro*)* in the general context of biocompatibility of materials for medical applications including HC [12]. In early seminal papers by Vroman and Adams [29, 30], interesting phenomena of competitive protein exchange on artificial surfaces have been observed, in which proteins already adsorbed on a surface (from a protein-mixture solution) are displaced by others, subsequently arriving. Significant research has followed because such exchanges, commonly referred to as the “Vroman effect”, seem to be related to blood platelet adhesion to surfaces and clotting (e.g. [31]). Of particular interest to this review are observations that, during the initial adsorption on surfaces, unfolding occurs of albumin and fibrinogen, under high concentrations as in HC [32]. Moreover, there is evidence that these proteins tend to competitively displace other adsorbed proteins [19, 33]. Soderquist and Walton [34] investigated the interrelation of adsorption/desorption processes (on co-polypeptide and silicone surfaces) with the structural changes of adsorbed albumin, y-globulin and fibrinogen. They suggested a three-stage process, including an initial reversible adsorption, a second phase where the adsorbed proteins undergo slow conformational change (with proteins essentially irreversibly adsorbed) and a final stage where the denatured material is slowly desorbed. The observed rather long timeframe of the last stage appears to be irrelevant to the shorter 4 h period of a HC session. It was also noted that denaturated albumin desorption (through such mechanism) or detachment by shear forces have been inadequately studied. Sivaraman and Latour [35] found that platelets bind to adsorbed albumin (through receptor-mediated processes), whose binding sites are formed by adsorption-induced protein unfolding. Importantly, a high degree of such unfolding, was correlated strongly with increased level of platelet adhesion. Moreover, greater albumin adsorption occurred with increasing albumin solution concentration**.** This was attributed to the fact that the transport rate of protein molecules to the surface increases as their concentration increases; thus, the molecules that adsorb from higher concentration have less time to unfold and spread before the surface becomes saturated with protein.

Pieniazek et al. [36] investigated changes in albumin structural characteristics during HD (haemodialysis). They evaluated the susceptibility of plasma albumin to oxidation in ESRD patients, before and after a HC session, in comparison to healthy persons. They also assessed the conformational state of albumin under such conditions, employing EPR (electron paramagnetic resonance) spectroscopy. Significantly, their data showed that during HC the level of thiols (± SH groups) was significantly affected, decreasing by ~ 15%. They concluded that the significant conformational changes, occurring in vivo during HC, negatively affect the albumin antioxidant function. Finally, Sishi et al. [37] recently investigated interactions between proteins and membrane material made of PES (polyethersulfone), PAN (polyacrylonitrile) and PVDF (polyvinylidene fluoride). In particular, they examined adsorption of main human serum proteins (albumin, fibrinogen, transferrin), at realistic concentrations, across the membrane thickness (i.e. into the pores), using an in-situ SR-μCT (Synchrotron-based X-ray micro-tomography) imaging technique. Albumin was preferentially adsorbed to all three membranes. PES membrane, possessing comparatively larger pores, adsorbs albumin within its whole thickness, whereas PAN and PVDF membranes tend to absorb it only at the top and in middle layers. SEM (scanning electron microscope) image analysis was employed to identify changes in the deposited proteins morphology, depending on membrane properties.

#### Studies on adsorbed /deposited albumin mass

There is a significant amount of in vitro work on protein adsorption and deposition to membranes, where HC conditions are simulated to various degrees, aiming to clarify the complicated phenomena involved, which will be briefly reviewed. On the contrary, there is hardly any definitive in vivo study regarding albumin mass adsorbed/deposited in HC filters.

Interpreting data on membrane performance, in an early clinical study, Rockel et al. [13] recognised the secondary membrane formation and its significant effects on permeability and species rejection, but did not determine the deposited-protein mass/loss. The latter was neglected and account was taken only of albumin leaking into the dialysate, i.e. ~ 1.4 g per session. Later Gachon et al. [38]**,** using an elution protocol, determined the adsorbed proteins on used HC filters after a session. The reported amount of adsorbed proteins, for ~ 1 m^2^ membrane surface area, was extremely small, i.e. < 10 μg total. The applied protocol involved extensive preliminary flushing (by recirculating saline solution), followed by sequential treatment with elution solutions; finally, reverse transmembrane pressure/flushing was applied to recover proteins adsorbed within the membrane pores. However, one can express reservations on the fitness of such protocol, to determine total mass of deposited proteins, particularly because the fouling layer (above the ‘tightly’ adsorbed proteins on the inner membrane surface) could be removed by flushing and be unaccounted. Significantly, the authors [38] express concern that protein may still remain adsorbed in the HC filters, even after the latter have been subjected to this intensive treatment protocol.

Langsdorf and Zydney [17] have shown that the permeation characteristics of particular flat-sheet (Cuprophan and PAN) membranes can be described using a two-layer membrane model, i.e. that a layer of adsorbed plasma proteins provides an additional resistance to mass transfer in series with that of membrane itself. Later, Morti and Zydney [39], using PAN and CTA (cellulose triacetate) HC filters, performed in vitro tests with human plasma, under rather “mild” conditions (*Q*_UF_ = 0, *Q*_blood_ = 200 and *Q*_dial_ = 500 mL/min) and measured permeability as well as other characteristics of deposited secondary layer. They determined experimentally the developing thickness of protein layer for PAN and CTA HC filters at 1.9 ± 0.5 μm and 4.4 ± 0.5 μm, respectively. It is estimated that, for a typical HC filter of 2.0 m^2^ surface area and fibre inner diameter 200 μm, a layer thickness 1.0 μm amounts to a deposit volume of ~ 2.0 mL (or ~ 2 g, for deposit density ~ 1 g/mL); therefore, the total mass of deposited proteins corresponding to these data is roughly ~ 4 g to ~ 9 g. However, under conditions of large convective/ultrafiltration rates (*Q*_UF_ ≈ 100 mL/min), as in OL-post HDF, one would expect a significantly greater mass of deposited proteins, including albumin. Birk et al. [40] tested (in vitro) 12 commercially available HC filters (using 11 different membrane materials) and perfused them with human blood containing 1251-labelled plasma proteins. Under filtration conditions (not quite representative of those prevailing in high-convection HC modes), the total protein adsorption ranged from 338 to 2098 mg/m^2^ membrane surface, whereas the fraction of adsorbed low-molecular-weight-proteins (LMWP < 65 kDa) varied between 14 and 70% of total protein.

Yamamoto et al. [16] investigated the effects of internal filtration/ultrafiltration on membrane fouling based on the membrane’s pure-water permeability, diffusive permeability, and sieving coefficient. Membrane fouling caused by protein adhesion was shown to increase due to enhanced ultrafiltration, particularly at the early treatment stage. Although evidence of membrane fouling was clear, the albumin/protein mass deposited on the membranes was not quantified. Tomisawa and Yamashita [41] using HC filters made of PMMA (polymethyl methacrylate) and PEPA (polyester polymer alloy) membrane material simulated HC by employing dilute synthetic BSA (bovine serum albumin) solutions (i.e. 5.10 g BSA in 2000 mL batches). It was reported that fractional BSA adsorption exceeded 50% (i.e. ~ 2.3 g) at rather high *Q*_UF_ (> 60 mL/min) with PMMA membranes but smaller with PEPA ones, concluding that the significant amount of albumin adsorbed by the membrane should be taken into account when a clinical criterion of the total albumin loss is considered. In vitro tests by Kim et al. [42] confirmed previous results [17] that protein deposition occurs quickly, noting that the properties of the protein-deposit become nearly constant after ~ 20 min as also noted earlier [17]*.* Moreover, it was suggested that protein deposition is enhanced by increasing ultrafiltration rates, further affecting HC performance.

Gomez et al. [22] employing a novel in vitro uremic matrix, determined total albumin loss during simulated HDF sessions. Reported data with a PMMA-HDF mode were: total mass of albumin extracted/lost (*M*_ext_) > 15 g, albumin lost in dialysate (*M*_dial_) ~ *5* g and albumin adsorbed only ~ 50 mg. The difference [*M*_ext_ − *M*_dial_] = > ~ 10 g may suggest that this albumin mass is absorbed/deposited within the module and possibly in the rest of HC circuit. This [*M*_ext_ − *M*_dial_] difference was ~ 4.5 g for CTA-HDF treatment*.* Although some sources of error or uncertainties should be considered, these relatively large albumin deposited/adsorbed mass cannot be overlooked. Kiguchi et al. [43] used dilute aqueous albumin solution (4 g/L), recirculated through three types of PEPA and one PSf (polysulfone) HC filter, to simulate fouling and study-related clearance effects. Although an albumin layer was developed and immobilised, the deposited albumin mass was not determined. In a clinical study, by Vanommeslaeghe et al. [44], involving 10 ESRD patients, data on albumin concentration in inlet and outlet blood/plasma streams were obtained (designated as Alb_inlet_, Alb_outlet_). These data were apparently used to correct respective venous concentrations, and determine extraction ratios, but not to estimate total albumin losses during HC session. Finally, Abdelrasoul et al. [45] investigated the competitive adsorption (on PES membrane) of main proteins albumin, fibrinogen (FB) and transferrin (TRF), by employing synthetic single and multiple protein solutions. In general, the proportion of adsorbed FB and TRF in the deposit was significantly greater than that in the initial protein feed-solution, suggesting preferential adsorption of those proteins compared to albumin. In addition, using the special SR-μCT technique, the adsorbed albumin within the membrane pores appeared to be dominant and substantial. However, no specific quantitative data on total adsorbed/deposited mass of those proteins were obtained, although recognised as significant.

There are few recent reviews on protein/membrane–material interactions of relevance to the topic of this paper. Huang et al. [14] dealt with blood–membrane interactions that influence solute removal. The role of secondary membrane formation, and concentration polarisation on membrane performance was discussed. Attention was paid to the composition of fouling layer (comprised mostly of the dominant proteins, albumin, fibrinogen, immunoglobulinG) and its effect on inflammatory response and thrombogenicity. Westphalen et al. [46] assessed our understanding protein-adsorption phenomena during HC, including related mechanisms and blood activations as well as the associated consequences. It was concluded that there is no model available to correlate/estimate the rate (or mass) of protein adsorption or the total amount of protein adsorbed during hemodialysis as a function of main operating conditions.

## Discussion

The research efforts to determine the total albumin losses during HC are part of the significant general efforts to better understand the effects of extracorporeal blood filtration, in various types of patient treatment including HC. In this context, it is desirable to develop sound criteria (applicable in medical practice) for implementing particular HC modes. Furthermore, it is expedient to give priority to such criteria for HC modes which are associated with the most severe conditions leading to the greatest albumin losses. OL-post HDF is broadly considered to be such a mode [2, 15]. The preceding review suggests that there are three types of potential losses, whose contribution to the total losses is unclear, primarily due to our incomplete physical understanding and the complexity of factors determining/causing them, as summarised in the following.

*Albumin permeating/‘leaking’ into the dialysate* is broadly recognised as a primary type of loss. As explained, it is relatively easy to determine in vivo*,* mainly through sample analyses and rather simple mass balance calculations on the disposable dialysate; thus, reliable data exist in the literature, obtained under various conditions (e.g. [7, 24–26]). Furthermore, the temporal variation of albumin sieving coefficient is broadly employed to characterise the performance of a particular HC mode and of the membrane used, in respect of targeted toxins removal. As is well known [2, 14, 15, 17, 26], albumin ‘leaking’ is significantly affected by the protein adsorption/deposition into the pores and onto the hollow-fibre inner surface, which reduce the permeability thus impacting on the HC membrane performance. Albumin leaking is reflected in the temporal variation of the respective sieving coefficient. However, it must be stressed that this variation (i.e. the ‘kinetics’ of albumin permeating the membrane), although it is an indicator of HC filter performance, should not be used to infer (or characterise) the development of fouling/“secondary”-membrane formation during HC, as is occasionally done (e.g. [28]). Importantly, based on this review, the use of albumin ‘leaking’ data to set criteria for the maximum permissible *total* albumin losses is considered unwarranted and questionable, as long as the other two types of losses are not quantitatively determined and comparatively assessed.

*Albumin loss by adsorption/deposition*. Quite a few in vitro studies (e.g. [22, 39–41]) strongly suggest the significant mass/loss of adsorbed/deposited albumin, of magnitude comparable to, or even greater than, that due to leakage. However, these results have not been confirmed by the existing meagre data from studies in vivo or by ex situ examination of HC filters after patients’ treatment. Regarding losses due to adsorption/deposition, one may differentiate between: (i) adsorption into the membrane pores and initial coverage by tightly bound proteins, (directly) on the inner membrane surface [37] and (ii) possible further ‘gel’/fouling-layer formation (beyond the initial surface layer) through copious protein/albumin deposition [35]. This distinction of the two sub-cases is seldom discussed in the HC literature [35]. The former type (i) is mainly responsible for the reduction of membrane permeability in the early stages of HC [17, 26, 42]. It may be added here that the gel/fouling layers (type ii) have been rather extensively studied in other ultrafiltration operations, treating much simpler dilute aqueous BSA solutions (e.g. [47]). It is shown there that for low permeation fluxes (in L/m^2^h), as in HC, such gel layers are not particularly coherent and their specific resistance to permeation is relatively low, which is in line with the observed quite small permeability reduction of HC membranes at long times (i.e. > 1 h) [17].

It should be stressed that high-convection modes (particularly OL-post HDF) favour ‘secondary membrane’ formation (particularly type ii above). Indeed, favourable conditions for a gel/fouling-layer formation are due to the imposed high ultrafiltration rate, which leads to increased albumin/protein concentration, plasma viscosity and polarisation phenomena as well as reduced axial shear stresses, along the entire length of the HC filter [1, 2, 15]. However, as outlined in “Issues regarding determination of albumin losses in vivo”, experimental difficulties and uncertainties are encountered to determine this type of deposits (i.e. adsorbed albumin and gel layer) by tests in vivo*,* particularly through blood/fluid sampling; thus, no such data exist. Rather limited data are available on the adsorbed albumin, by employing ex situ elution techniques on used modules (i.e. [38]). Importantly, these protocols (involving rather intensive preliminary flushing and subsequent elution) likely remove the ‘gel’ layer together with all the proteins and other species remaining within the used HC filter, including protein mass in the filter entry and exit sections as well as in the rest of the extracorporeal circuit; however, the latter are essentially losses to be accounted for. Moreover, problems and uncertainties related to the adequacy of elution techniques are occasionally reported (e.g. [38]). Therefore, these elution protocols tend to identify and possibly quantify only the initial tightly bound surface layer (type i above). Evidently, appropriate experimental protocols need to be developed/improved to permit reliable determination of the total adsorbed/deposited albumin mass.

*Albumin conformational changes* (i.e. leading to denatured albumin in flowing plasma), which impair the normal/natural albumin functions, should be also viewed as a loss. Conformational changes have been recently identified in characterisation/analysis of ESRD patients’ blood before and after HC [36]; such changes may be attributed [at least partly] to the dynamics of blood–material interactions which are well documented in in vitro studies (e.g. [19, 31]. Contact with other components (e.g. pumps, piping) of the extracorporeal circuit might also contributes to such albumin conformational changes. No particular attention is currently paid in the literature to quantify such effective losses. Moreover, the difficulty to quantitatively determine the albumin losses due to denaturation, from in vivo data, may be also (at least partly) due to the fact that the commonly used methods of blood characterisation (based on dye binding, size exclusion, and immunoassay techniques) cannot distinguish between native and denatured albumin [48].

The preceding review clearly suggests that priority in research efforts should be given to clarify blood/plasma interactions with the hollow-fibre membranes (and possibly with other components of the HC extracorporeal circuit), thus allowing to quantify the albumin losses, separately, due to adsorption/deposition and denaturation. Only then sound criteria on maximum permissible *total* albumin losses during HC can be developed. In addition, development of facile and accurate methods to determine (separately) the native and denatured albumin concentration, in the context of in vivo tests, would greatly aid these efforts. The suggested specific research targets can be pursued in the context of significant ongoing efforts aiming to improve biocompatibility of materials towards optimisation of HC modes [11, 12].

## Concluding remarks

This review suggests that, during haemocatharsis of ESRD patients, three types of potential albumin losses can contribute to the total amount lost per session, i.e. losses due to (i) albumin ‘leaking’ into the permeate, (ii) membrane fouling (or ‘secondary’ membrane formation) and (iii) conformational albumin changes of the treated blood/plasma, essentially depriving albumin of its natural functions. Reliable data exist on the first type of losses, and can be obtained in vivo rather easily. In respect of albumin adsorption/deposition losses, very meagre quantitative information exists from in vivo studies, despite their well-known key role in HC performance; lack of such data is mainly attributed to experimental difficulties. Finally, the quantitative determination of albumin conformational changes (i.e. effectively ‘losses’) has been essentially neglected (with notable recent exceptions), despite extensive work on their possible negative role in triggering undesirable effects (e.g. complement activation, platelet adhesion, reduced antioxidant function, blood clotting).

Based on this critical review, the use of albumin ‘leaking’ data (as a sole quantifiable type of losses), to set criteria for the maximum permissible total albumin losses, is considered unwarranted and questionable, as long as the other two types of losses have not been quantitatively determined and comparatively assessed. Obviously, research efforts should focus on better understanding and quantifying albumin losses due to adsorption/deposition and denaturation.

## References

[1] Ronco C, Clark WR (2028). Haemodialysis membranes. Nat Rev Nephrol.

[2] Canaud B (2021). Recent advances in dialysis membranes. Curr Opin Nephrol Hypertens.

[3] Lang T, Zawada AM, Theis L, Braun J, Ottillinger B, Kopperschmidt P, Gagel A, Kotanko P, Stauss-Grabo M, Kennedy JP, Canaud B (2023). Hemodiafiltration: technical and medical insights. Bioengineering.

[4] Ward RA, Beck W, Bernardo AA, Alves FC, Stenvinkel P, Lindholm B (2019). Hypo-albuminemia: a price worth paying for improved dialytic removal of middle-molecular-weight uremic toxins?. Nephrol Dial Transplant.

[5] Saito A (2011). Definition of high-performance membranes—from the clinical point of view. Contrib Nephrol.

[6] Boschetti-de-Fierro A, Beck W, Hildwein H, Krause B, Storr M, Zweigart C (2017). Membrane innovation in dialysis. Contrib Nephrol Karger.

[7] Potier J, Queffeulou G, Bouet J (2016). Are all dialyzers compatible with the convective volumes suggested for postdilution online hemodiafiltration?. Int J Artif Organs.

[8] van Gelder MK, Abrahams AC, Joles JA, Kaysen GA, Gerritsen KGF (2018). Albumin handling in different hemodialysis modalities. Nephrol Dial Transplant.

[9] Kalantar-Zadeh K, Ficociello LH, Bazzanella J (2021). Slipping through the pores: hypoalbuminemia and albumin loss during hemodialysis. Int J Nephrol Renovasc Dis.

[10] Bowry SK, Kircelli F, Himmele R, Nigwekar SU (2021). Blood-incompatibility in haemodialysis: alleviating inflammation and effects of coagulation. Clin Kidney J.

[11] Zawada AM, Lang T, Ottillinger B, Kircelli F, Stauss-Grabo M, Kennedy JP (2022). Impact of hydrophilic modification of synthetic dialysis membranes on hemocompatibility and performance. Membranes.

[12] Ji H, Li Y, Su B, Zhao W, Kizhakkedathu JN, Zhao C (2023). Advances in enhancing hemocompatibility of hemodialysis hollow-fiber membranes. Adv Fiber Mater.

[13] Rockel A, Hertel J, Fiegel P, Abdelhamid S, Panitz N, Walb D (1986). Permeabillity and secondary membrane formation on a high flux polysulfone hemofilter. Kidney Int.

[14] Huang Z, Gao D, Letteri JJ, Clark WR (2009). Blood-membrane interactions during dialysis. Semin Dial.

[15] Pstras L, Ronco C, Tattersall J (2022). Basic physics of hemodiafiltration. Semin Dial.

[16] Yamamoto K, Hiwatari M, Kohori F, Sakai K, Fukuda M, Hiyoshi T (2005). Membrane fouling and dialysate flow pattern in an internal filtration enhancing dialyzer. J Artif Organs.

[17] Langsdorf LJ, Zydney AL (1994). Effect of blood contact on the transport properties of hemodialysis membranes—a 2-layer membrane model. Blood Purif.

[18] Bosch T, Schmidt B, Samtleben W, Gurland HJ (1985). Effect of protein adsorption on diffusive and convective transport through polysulfone membranes. Contrib Nephrol.

[19] Hirsh SL, McKenzie DR, Nosworthy NJ, Denman JA, Sezerman OU, Bilek MM (2013). The Vroman effect: competitive protein exchange with dynamic multilayer protein aggregates. Colloids Surf B.

[20] Brash JL, Horbett TA, Latour RA, Tengvall P (2019). The blood compatibility challenge Part 2: protein adsorption phenomena governing blood reactivity. Acta Biomater.

[21] Melchior P, Erlenkötter A, Zawada AM, Delinski D, Schall C, Stauss-Grabo M (2021). Complement activation by dialysis membranes and its association with secondary membrane formation and surface charge. Artif Organs.

[22] Gomez M, Bañon-Maneus E, Arias-Guillén M, Maduell F (2020). Assessment of removal and adsorption enhancement of high-flux hemodialyzers in convective therapies by a novel in vitro uremic matrix. Sci Rep.

[23] Gayrard N, Ficheux A, Duranton F, Guzman C, Szwarc I, Vetromile F, Cazevieille C, Brunet P, Servel M-F, Argiles A, Le Quintrec M (2017). Consequences of increasing convection onto patient care and protein removal in hemodialysis. PLoS One.

[24] Tsuchida K, Minakuchi J (2011). Albumin loss under the use of the high performance membrane. Contrib Nephrol.

[25] Fournier A, Birmele B, Francois M (2015). Factors associated with albumin loss in post-dilution hemodiafiltration and nutritional consequences. Int J Artif Organs.

[26] Vega A, Quiroga B, Abad S, Aragoncillo I, Arroyo D, Panizo N, López-Gómez JM (2015). Albumin leakage in online hemodiafiltration, more convective transport, more losses?. Ther Apher Dial.

[27] Cuvelier C, Tintillier M, Migali G, Van Ende C, Pochet J-M (2019). Albumin losses during hemodiafiltration: all dialyzers are not created equal—a case report. BMC Nephrol.

[28] Ehlerding G, Ries W, Kempkes-Koch M, Ziegler E, Erlenkötter A, Zawada AM, Kennedy JP, Ottillinger B, Stauss-Grabo M, Lang T (2022). Randomized comparison of three high-flux dialyzers during high-volume online hemodiafiltration—the comPERFORM study. Clin Kidney J.

[29] Vroman L, Adams AL (1969). Findings with recording ellipsometer suggesting rapid exchange of specific plasma proteins at liquid/solid interfaces. Surf Sci.

[30] Vroman L, Adams AL (1969). Identification of rapid changes at plasma-solid interfaces. J Biomed Mater Res.

[31] Vroman L, Adams AL, Fischer GC, Munoz PC (1980). Interaction of high molecular weight kininogen, factor-xii, and fibrinogen in plasma at interfaces. Blood.

[32] Roach P, Farrar C, Perry CC (2005). Interpretation of protein adsorption: surface induced conformational changes. J Am Chem Soc.

[33] Elgersma AV, Zsom RLJ, Lyklema J, Norde W (1992). Adsorption competition between albumin and monoclonal immunogammaglobulins on polystyrene lattices. J Colloid Interface Sci.

[34] Soderquist M, Walton A (1980). Structural changes in proteins adsorbed on polymer surfaces. J Colloid Interface Sci.

[35] Sivaraman B, Latour RA (2010). The Adherence of platelets to adsorbed albumin by receptor mediated recognition of binding sites exposed by adsorption induced unfolding. Biomaterials.

[36] Pieniazek A, Gwozdzinski L, Zbrog Z, Gwozdzinski K (2018). Alterations in conformational state of albumin in plasma in chronic hemodialyzed patients. PLoS One.

[37] Sishi Z, Bahig J, Kalugin D, Shoker A, Zhu N, Abdelrasoul A (2023). Influence of clinical hemodialysis membrane morphology and chemistry on protein adsorption and inflammatory biomarkers released: In-situ synchrotron imaging, clinical and computational studies. Biomed Eng Adv.

[38] Gachon AMF, Mallet J, Tridon A, Deteix P (1991). Analysis of proteins eluted from hemodialysis membranes. J Biomater Sci Polym Ed.

[39] Morti SM, Zydney AL (1998). Protein-membrane interactions during hemodialysis: effects on solute transport. ASAIO J.

[40] Birk H-W, Kistner A, Wizernann V, Schiitterle G (1995). Protein adsorption by artificial membrane under filtration conditions. Artif Organs.

[41] Tomisawa N, Yamashita AC (2009). Amount of adsorbed albumin loss by dialysis membranes with protein adsorption. J Artif Organs.

[42] Kim TR, Hadidi M, Motevalian SP, Sunohara T, Andrew L, Zydney AL (2018). Effects of plasma proteins on the transport and surface characteristics of polysulfone/polyethersulfone and asymmetric cellulose triacetate high flux dialyzers. Artif Organs.

[43] Kiguchi T, Tomisawa Ν, Akihiro C, Yamashita AC (2022). Replication of fouling in vitro in hollow fiber dialyzers by albumin immobilization. J Artif Organs.

[44] Vanommeslaeghe F, Josipovic I, Boone M, Van Biesen W, Eloot S (2022). Impact of intradialytic fiber clotting on dialyzer extraction and solute removal: a randomized cross over study. Sci Rep.

[45] Abdelrasoul A, Zhu N, Doan H, Shoker A (2023). In-situ synchrotron quantitative analysis of competitive adsorption tendency of human serum proteins on polyether sulfone clinical hemodialysis membrane. Sci Rep.

[46] Westphalen H, Abdelrasoul A, Shoker A (2021). Protein adsorption phenomena in hemodialysis membranes: mechanisms, influences of clinical practices, modeling, and challenges. Colloid Interface Sci Commun.

[47] Sioutopoulos D, Karabelas AJ, Mappas V (2019). Membrane fouling due to protein-poly-saccharide mixtures in dead-end ultrafiltration; the effect of permeation flux on fouling resistance. Membranes.

[48] You L, Wang X, Wang W (2021). Novel substrate-inspired fluorescence-based albumin detection improves assessment of clinical outcomes in hemodialysis patients receiving a nursing nutrition intervention. Med Sci Monit.

